# The Illusory Case for Treatment of an Invented Disease

**DOI:** 10.3389/fendo.2021.682620

**Published:** 2022-01-18

**Authors:** David J. Handelsman

**Affiliations:** ^1^ ANZAC Research Institute, University of Sydney, Sydney, NSW, Australia; ^2^ Andrology Department, Concord Hospital, Sydney, NSW, Australia

**Keywords:** testosterone, ageing, sexual dysfunction, fertility, andropause

## Introduction

Debates are usually understood as contests between two opposing cases, each argued with reasonable *a priori* plausibility. However, this debate on therapeutics is based on a false premise. The onus for justifying a new, non-approved prescription treatment lies unilaterally with proponents to establish a convincing case for efficacy and safety in treating a specific disease. It does not rest with the unconvinced to rebut such presumptions. While this onus is clearly defined in legal terms for commercial sponsors seeking marketing for a drug, the logic is equally true for enthusiasts promoting a novel medical treatment. In this case, asserting a justification for testosterone treatment for male ageing fails on all three counts - unconvincing efficacy, unproven and doubtful safety for the mirage of an invented “disease”. This review will outline the scant, inconclusive evidence for efficacy and safety while highlighting the invented “disease” is poorly defined, motivated by vague, subjective, wishful thinking about rejuvenation. Yet experience shows that once ingrained by simple repetition supplemented by confirmation bias, such an abstract illusion becomes reified into unshakeable delusion, impervious to evidence. This combination of circumstances has culminated in a quasi-epidemic increase in testosterone prescribing over three decades evident in the 100-fold increase in testosterone prescription sales by 2010 (1), an upsurge without any new approved indications. Such massive overuse of testosterone reflects its phenomenal pre-scientific popularity (including among doctors) as an attractive but unproven anti-ageing tonic for the loss of energy or vitality as well as sexual dysfunction, resting on wishful thinking about the mirage of rejuvenation rather than sound evidence. This popularity revolves around testosterone perceived as an elixir of youth, a fantasy of rejuvenation requiring minimal marketing to a receptive audience. Virtually every hormonal system displays age-related changes that may be viewed from the framework of rejuvenation as impairments susceptible to hormone replacement therapies. Yet the late 20^th^ century revival of the rejuvenationist imaginings from the turn of that century (see below) has focused exclusively on testosterone, reflecting communal perceptions of testosterone as an elixir of youthful vigour. Above all the successes of medicine in the 20^th^ century reaffirm that legitimate medical therapies must focus on a specific, well defined organic disorder so that the requisite clinical trials for efficacy and safety have a firm foundation. That is not, nor ever likely to be, true for medicalizing of ageing itself and remains contentious for the tunnel-visioned surrogates such as reduced blood testosterone without regard to underlying systemic diseases or definitive linkage to symptoms.

## The Invention of Ageing as a Treatable “Disease”

***Even when history does not exactly repeat itself, it usually rhymes (Mark Twain) but what we learn most from history is that we do not learn from history (GWF Hegel).***

This present epidemic of excessive testosterone prescribing recapitulates the wishful thinking about rejuvenation from the era of rejuvenation quackery, known as organotherapy, at the turn of the 20^th^ Century (2). Ushered in by Charles-Edouard Brown-Sequard’s 1887 announcement that self-injection of his aqueous extract of animal testes produced miraculous, prolonged revival of his mental and physical health (3). Derided by his professional transatlantic colleagues as fantasy (4–6), this was unquestionably a placebo response because his well-recorded method of an aqueous testicular extract contained virtually no hydrophobic steroids such as testosterone (7). Yet, based on his report, *methode sequardienne* became a raging commercial success for male rejuvenation till it, as well as its me-too alternative methods by Steinach and Voronoff, disappeared abruptly in the 1930s (8). That disappearance coincided with the Great Depression removing discretionary spending on frivolous ephemeral hobbies together with the discovery of testosterone as the testicular male sex hormone removing organotherapy’s façade of scientific credibility. Historically, however flamboyant, this episode was not isolated nor the first eruption of rejuvenation fantasies, which re-emerge whenever sufficient societal affluence allows for indulgence in the wishful thinking about the hobby of life extension. Another famous episode occurred around 16^th^ century expeditions of Juan Ponce de Leon to the Caribbean which landed in Florida in search of the fabled Fountain of Youth, legendary spring waters which magically restore youthfulness (9). This voyage, possibly as a lucrative sideline to piracy, was sponsored by the King of Spain who had recently married a woman 35 years his junior.

Modern medicine is founded on the pathological basis of disease for which treatments are designed to prevent, halt, reverse or cure organic disease. This postulates that medical diseases have an organic basis, that prescription drug treatments should be related to the underlying pathophysiological mechanisms and must provide convincing evidence of efficacy and safety for licensing as prescription medicines. Within this framework, drug prescription is a monopoly granted to qualified, registered doctors, a privilege maintained by the responsible exercise of that right through scrupulous, knowledgeable, and ethical medical practice. While that right extends to prescribing drugs for off-label indications, that liberty should be exercised based on sound, unconflicted medical judgement and evidence. Systematic violation of responsible prescribing can lead to individual doctors being deregistered and, more broadly, to undermining the social licence for doctors to maintain their monopoly on drug prescription which, ultimately, could lead to regulatory narrowing such as restricting prescribing to approved indications. Hence, modern therapeutics is tied to preventing, treating, or curing authentic disease rather than simply using drugs on demand, or for pleasure or illusory benefits and that includes inventing new “diseases” to mask drug misuse or abuse.

**Figure 1:**
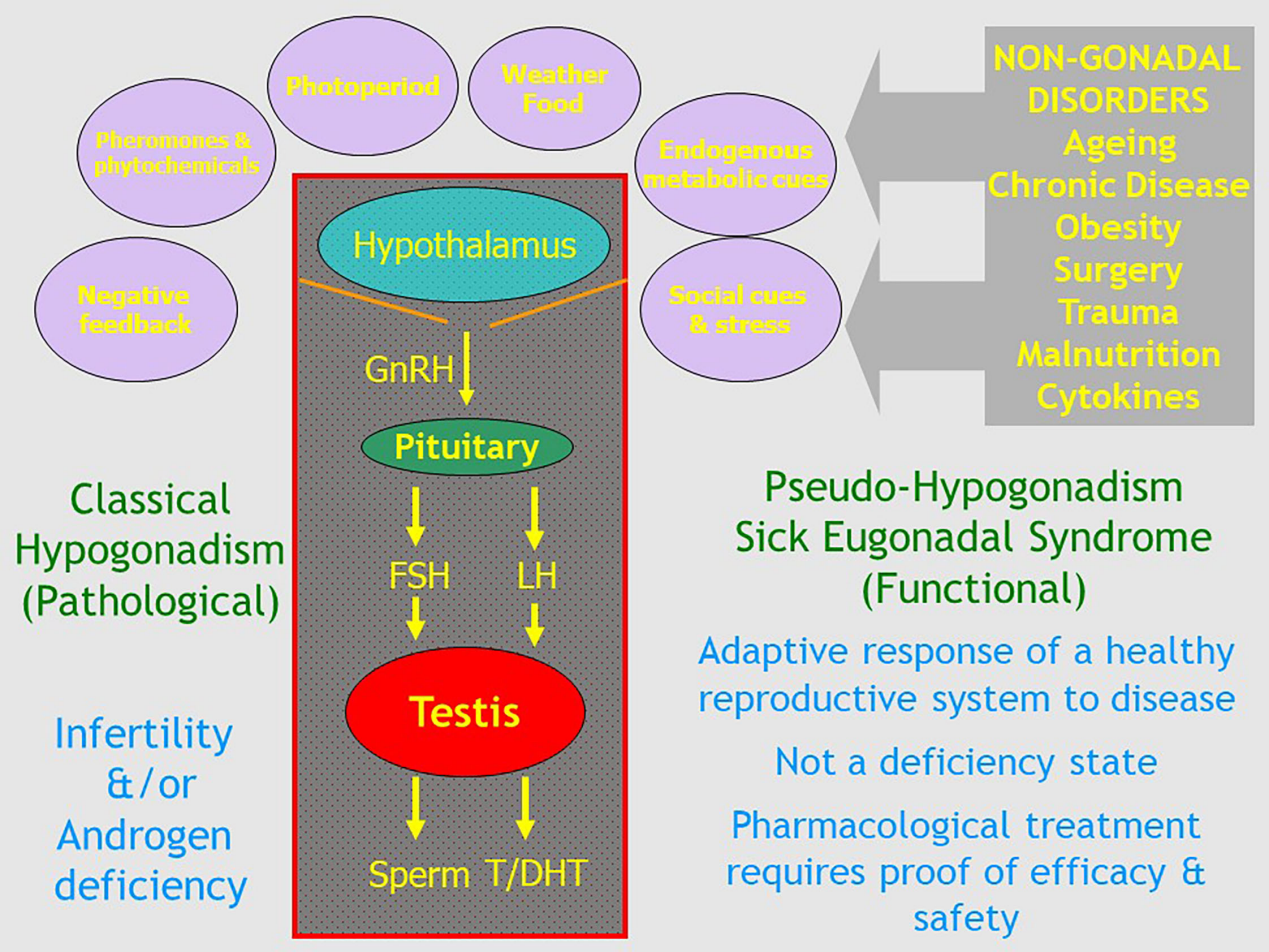
Schematic outline of the relationship of classical pathologic hypogonadism due to irreversible organic disorders of the hypothalamo-pituitary testicular axis with functional Pseudo-Hypogonadism or Sick Eugonadal Syndrome which reflects the potentially reversible impact of exogenous environmental and internal cues mediated by the hypothalamus as a transducer of signals that govern the overall activity of the male reproductive axis.

In this context, terminology is important. Clear, cogent discussion is impeded when conducted in terms that are confused, confusing or ambiguous. Therapeutics must start with a well-defined disease. For male ageing, there is no objective and reproducible basis for classifying it as a treatable disease per se, with the medicalizing of ageing simply an illusory pursuit. One evasion of this logical *cul de sac*, to legitimize this misdirected goal, is to disguise it by redefining ageing to give it a more credible sounding medical or pathological basis. Thus, the medicalising of male ageing has been embodied in the entity of “age-related hypogonadism”, the most neutral of the plethora of terms (see below) introduced to give this drive an upmarket medical gravitas. The key to this process is redefining the term “hypogonadism”. In men, this term has always been defined as pathological disorders of the reproductive system including organic disease of the testis (primary hypogonadism) and the hypothalamus or pituitary (secondary hypogonadism) known collectively as the hypothalamo-pituitary testicular (HPT) axis. These disorders impair the twin functions of the testis - steroidogenesis and spermatogenesis – possibly leading to androgen deficiency and subfertility, respectively (**Figure 1**). But in recent years this conventional definition has been hijacked by disease mongering, the elastic widening of the boundaries of treatable disorders through redefining the target disease with the pharma industry goal and net effect of promoting more drug prescribing (10–12). This modern elastic redefinition of “hypogonadism” broadens its boundaries to include any lowering of serum testosterone in one or more blood samples coupled with any of a long list of non-specific clinical features (13). This expansive redefinition lacks proper focus on underlying reproductive disorders and substitutes a simpler focus on coupling virtually any symptomatic complaint with a lowered blood testosterone while also ignoring spermatogenesis. This adopts the simplistic, narrowed interpretation that any reduction in blood testosterone must cause non-specific symptoms rather than reverse causality (that underlying disorders cause reduction in blood testosterone) or that both the symptoms and lower testosterone represent effects in common of an underlying disorder.

To restate the classical definition, hypogonadism is a clinical diagnosis with a pathological basis, confirmed by hormone assays – not the other way around (2). The latter misconception of a low circulating testosterone as a diagnostic fulcrum is the basis of most (13–17), but not all (18), current clinical guidelines on diagnosis and management of hypogonadism. That majority of opinions pivot the diagnosis of “hypogonadism” on potentially reversible functional causes of a low circulating testosterone rather on irreversible pathological causes. Effectively, this pathologizes ageing and/or obesity by deeming them causes of “hypogonadism”. By begging the question and falsely invoking a hormone deficiency state, this approach purports to bypass the need for sound evidence-based evidence of efficacy and safety for testosterone treatment of functional states having in common only low circulating testosterone without regard to its basis. This confected rationalization of otherwise unjustified testosterone treatment is undoubtedly an important engine of the epidemic of overprescribing of testosterone in the last few decades. 

Another important consequence of the misdirected focus on testosterone in male ageing is the proliferation of medical screening for low testosterone. Given such direct screening is not recommended for lack of data by even the most enthusiastic of guidelines, this is sometimes deceptively adopted when referred to as “case finding”, simply a personalised form of medical screening. Typically, such screening is undertaken by measuring blood testosterone in isolation prompted by almost any presenting complaint. Then when blood testosterone is considered low, this is labelled as “hypogonadism” representing an indication to commence testosterone treatment. Devoid of proper consideration of concomitant systemic disorders, this narrow screening-type approach is usually not accompanied by measuring blood LH, FSH and SHBG, biomarkers indispensable to properly evaluate androgen status. The simplistic screening for low testosterone approach, once fostered overtly by pharma (“LowT” campaigns) (19, 20) together with upscale single-issue health clinics (21), although these now replaced by more sophisticated marketing approaches obscuring vested commercial interests. In concert they encourage the overdiagnosis of “hypogonadism” responsible for the quasi-epidemic of excessive, unjustified testosterone prescribing. Proper consideration of androgen status requires at least multiple blood samples including measurement of serum SHBG to qualify interpretation of blood testosterone measurement. For example, the low blood SHBG in obese men creates an expectation of a comparably low blood testosterone, without signifying an androgen deficiency state. Additionally, concurrent measurement of blood LH and FSH allows evaluation of net androgen status for men with normal hypothalamic function in whom they operate as androgen sensors (22). Finally, measurement of bone density also contributes to an evaluation of net androgen exposure over time. The characteristic hormonal features of obese men, low blood testosterone and SHBG with normal LH and FSH, indicate a eugonadal state, better termed pseudo-hypogonadism. A complete description of the appropriate diagnostic workup for a diagnosis of hypogonadism is beyond the scope of this review but is available elsewhere (18, 22, 23).

Modern marketing of androgen misuse requires a form of social branding to legitimize off-label testosterone prescribing in the absence of proven indications or sound evidence of efficacy and safety. For this purpose, inventing an attractive and popular terminology has attracted significant efforts with the espousal of synonyms including “male menopause”, “climacteric”, “andropause”, “viropause”, **“(partial) androgen deficiency in the aging male [(P)ADAM]**, “LowT”, and the most recent, upmarket makeover to create greater medical gravitas, “late-onset hypogonadism”. The terminology adopted in the title of this debate “late-onset hypogonadism” is only among the latest descriptors of a series of neologisms for what may be most neutrally described [e.g. by FDA (24)] as a state of age-related reduced blood testosterone. The motivation for this drive is apparent from the known prevalence of androgen deficiency due to pathological hypogonadism [~0.5% of men (22)] compared with the estimated prevalence of “andropause” as up to 40% (25), or more usually claimed in the range 10-25% (26–28), and even the most modest estimates of 2-3% (29) representing major (5-100 fold) increases in potential market size over pathological hypogonadism. Furthermore, there is a compelling commercial logic for naming age-related reduction of blood testosterone. Creating a memorable name for an invented entity creates a reality and a thereby therapeutic target. By hijacking the term “hypogonadism” to create an expanded definition of the term, this purports to circumvent the need to prove efficacy and safety for such testosterone treatment as it purports to be an acceptable use of testosterone replacement therapy for pathologic hypogonadism (30). Moreover, this tactic turns the logical tables on the unconvinced who are then posed with the impossibility of trying to proving a negative, the nonexistence of the newly invented entity. As both an example and counterexample at once, such creation of a memorable name for an invented entity might be known as the Unicorn syndrome.

Crucially, the expansive redefinition of “hypogonadism” ignores the lack of evidence that the circulating testosterone and symptoms attributed to it are in fact linked causally. Even if they are, the direction of causality cannot be deduced from observational data. For example, in the most ambitious attempt to define “late-onset hypogonadism”, the EMAS observational cohort study of over 3300 men from 8 European countries reported that lowered blood testosterone was not significantly linked to any of a wide variety of psychological or physical symptoms but had a weak inverse correlation with three sexual dysfunction symptoms (31). However, the unquestioned interpretation in that study (and many others) that mildly reduced blood testosterone caused the sexual dysfunction symptoms ignores the likelihood of reverse causality. Accumulating evidence, however, indicates that sexual activity maintains blood testosterone so that reduced sexual activity is expected to reduce blood testosterone (32–36). Hence mild lowering of blood testosterone is likely an expected consequence, rather than a cause of, sexual dysfunction in male ageing. In effect, these issues nullify the proposed definition of “late-onset hypogonadism” and its neologistic synonyms.

Simplistic misinterpretation of a lowered blood testosterone in isolation as “hypogonadism” is most frequently encountered by misunderstanding the reduced blood testosterone in obesity and in the closely related type 2 diabetes mellitus (37, 38). In these conditions, blood testosterone is reduced proportionately to the lowering of blood SHBG and the excess body weight, with both causative effects reversed by effective weight reduction (38). The primary impact in lowering blood testosterone in obesity is through a reduction of blood SHBG, the carrier protein for the majority of circulating testosterone. That means that a reduced blood SHBG inevitably lowers blood testosterone proportionately (39). Invoking so-called “free” testosterone to evaluate androgen status in obesity only sows confusion with a logical circularity (2, 40, 41). Androgen status in obese men is best interpreted by the consistently normal serum LH and FSH. For men with normal hypothalamic function, blood gonadotrophins operate effectively as androgen sensors like TSH does for thyroid function. This interpretation of serum LH comes with the caveat that (a) pulsatility of circulating LH requires multiple samples and (b) serum LH lacks a well-defined lower limit of normal, in contrast to serum TSH by third generation immunoassay (42). Nevertheless, these hormonal features indicate that, in general, obesity is not an androgen deficiency state so that testosterone replacement is not warranted. The most extreme forms of obesity may have underlying hypothalamic disturbance (37) but for such patients bariatric surgery is the most appropriate treatment, not testosterone. This misinterpretation of obesity effects on blood testosterone in middle-aged men (then misdefined as “hypogonadism”) is among the major drivers of testosterone misuse (43–46). Obese men with non-specific symptoms are often screened biochemically by measuring blood testosterone in isolation, but without LH, FSH and SHBG to clarify interpretation. Any expected lowering of blood testosterone would then diagnose “hypogonadism” leading to pointless, ineffective testosterone treatment (37, 43–45). This characteristic hormonal pattern in obesity and type 2 diabetes – low blood testosterone and SHBG with normal LH and FSH - is better termed pseudo-hypogonadism.

In many other systemic disorders that accumulate as co-morbidities of male ageing, the impact of systemic illness on a normal HPT unit may lead to reduced testosterone secretion as part of a normal hypothalamo-pituitary response to adverse environmental conditions. That state, termed ontogenic regression, is a reversible partial reversion to the immature regulatory state of the reproductive system during pre-puberty. During adverse environmental conditions, whether endogenous or exogenous, the reproductive system undergoes orderly withdrawal using the mechanisms to return to the immature reproductive state of childhood. This orderly reproductive system regression has an inbuilt mechanistic opportunity for revival with the potential to revert to normal, if and when environmental adversity resolves. The important point is that such functional responses to systemic illness do not necessarily constitute an androgen deficiency state. Instead, as with the sick euthyroid or nonthyroidal illness syndrome (47), this may represent an adaptive conservative pathophysiological response such as temporary withdrawal of reproductive capacity during adverse environmental circumstances. Any use of testosterone whether for physiological replacement, or at higher doses for pharmacological androgen therapy, requires proof of efficacy and safety for that indication.

These effects of systemic illness on male reproductive function resemble those for thyroid function, known as the sick euthyroid or nonthyroidal illness syndrome (NTIS) (47–50). NTIS occurs in a wide variety of acute and chronic systemic illnesses where it may be an adaptive mechanism to save energy and/or activate immune mechanisms during severe illness, injury, or other catabolic states (47). Its features of a low blood triiodothyronine (T3) with increased reverse T3 without consistent changes in TSH or thyroxine (T4) reflect increasing peripheral metabolism of T3 with preferential shunting of T4 metabolism to rT3 modulated by cytokine and/or nutritional influences. Multiple randomized, placebo-controlled clinical trials of thyroid hormone (T3 and/or T4) administration have shown no consistent benefit. As a result, prolonged controversy [including a famous debate (51, 52)] has not provided support for NTIS being a state of hypothyroidism warranting thyroid hormone administration (47–49). The impact of ageing on male reproductive function where there is lowering of blood testosterone without consistent changes in LH or FSH may analogously be considered as a sick eugonadal or nonreproductive illness syndrome. Equally it does not warrant testosterone replacement or pharmacological androgen therapy, at least without convincing placebo-controlled clinical trial evidence of efficacy and safety.

More broadly, the decline in blood testosterone in male ageing is neither inevitable nor related to age per se. Rather as the accumulation of comorbidities of ageing, it is inherently at least partially avoidable or reversible by alleviation of those co-morbidities. For example, the Healthy Man Study showed that among 325 men studied 9 times over 3 months recruited for their excellent, symptom-free health there was no age-related decline in serum testosterone (or its metabolites dihydrotestosterone and estradiol) despite the study sensitive enough to detect changes from fasting, obesity and smoking (53). The interpretation that the age-related decline in blood testosterone is related to co-morbidities rather than ageing per se is supported by other longitudinal studies of reproductive hormones in male ageing (54, 55).

## Efficacy

**Figure 2:**
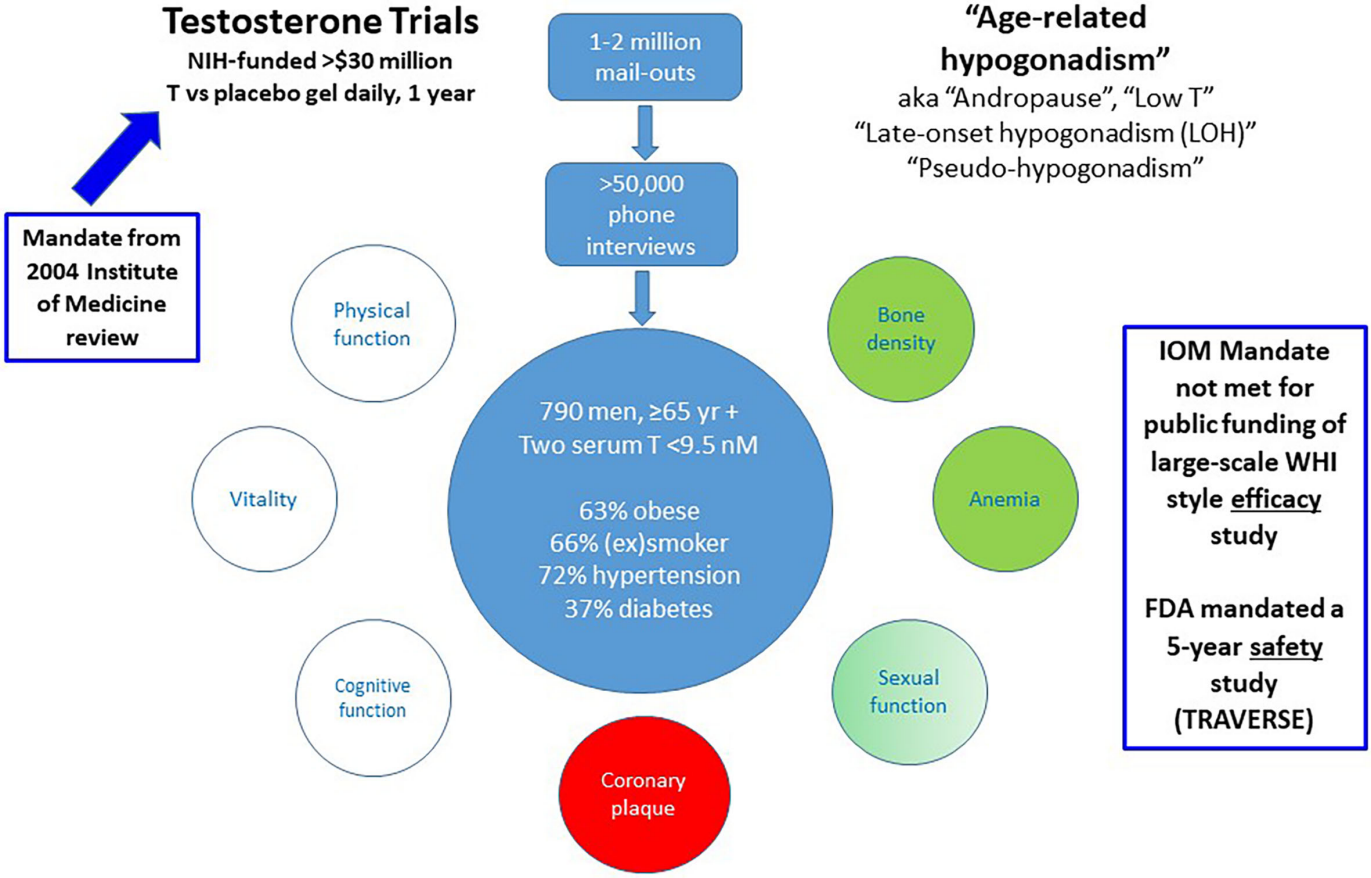
Summary of the US NIH-funded Testosterone Trials. These were prompted by the 2004 Institute of Medicine report leading to NIH funding of seven integrated placebo-controlled randomized clinical trials of transdermal testosterone treatment daily for 12 months to investigate potential efficacy benefits. Although Testosterone Trails did not meet the efficacy mandate of the IOM report to warrant public funding, the FDA mandated a pharmaceutical company sponsored 5 year safety study (TRAVERSE).

The strongest objective argument favouring testosterone treatment for male ageing is the modest, transient efficacy shown for improving sexual function together with increasing bone density and hemoglobin in the Testosterone Trials (56). However, the wider context of this study is important for its interpretation. The Testosterone Trials were prompted by the 2003 report by the independent Institute of Medicine (IOM, now National Academy of Medicine) (57). After reviewing available evidence for efficacy and safety of testosterone treatment for ageing men, the report concluded there was insufficient evidence of efficacy to warrant a publicly funded study comparable in scope to the Women’s Health Initiative (WHI) (58). Instead, they recommended a series of short-term studies of efficacy, a proposal funded by the NIH as the Testosterone Trials. This was a well-designed series of seven overlapping, integrated clinical trials that randomized 790 men over 65 without pathologic hypogonadism but with reduced blood testosterone to evaluate the effects of daily testosterone (*vs* placebo) transdermal gel over a 12-month trial (**Figure 2**). These studies reported transient and modest increase in sexual function - about a 1/3 increase over baseline sexual activity - which waned to non-significance by the end of the study (56, 59). Those benefits in sexual function were less effective than those of phosphodiesterase 5 inhibitor treatment in similar men. The study also reported small increases in bone density (60) and hemoglobin (61), changes expected of any androgen treatment in anyone. However, there were no benefits in physical activity, vitality, or cognitive function (56) and an unexpected and unprecedented increase in non-calcified coronary plaque growth (62, 63). Editorial commentaries indicated these modest, mixed efficacy benefits did not justify initiating testosterone treatment nor did they meet the mandate of the IOM report for justifying a public funding of a WHI-style clinical trial (64, 65).

**Figure 3:**
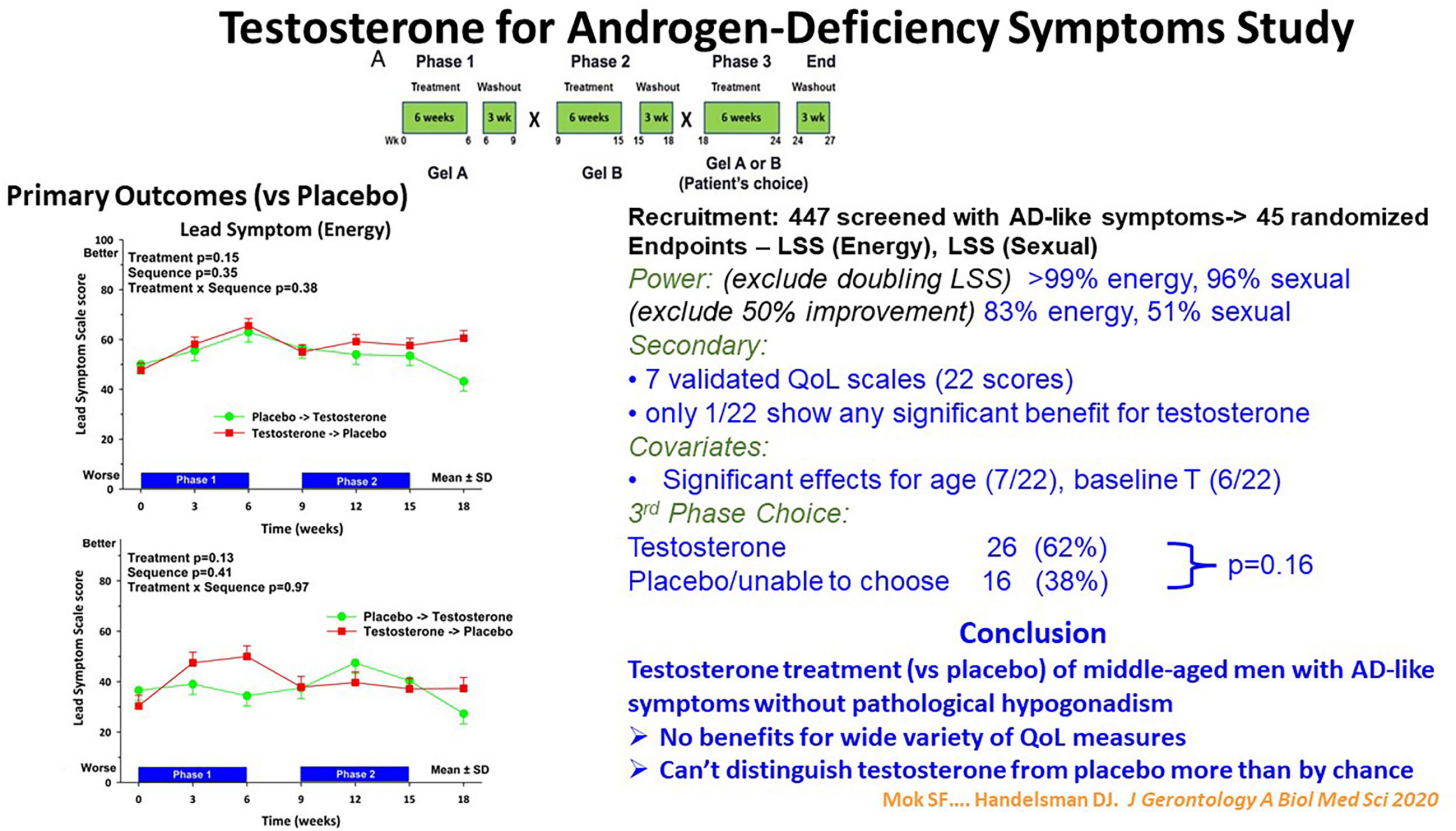
Summary of the Testosterone for Androgen-Deficiency Symptoms (TADS) Study, a randomized placebo-controlled cross-over clinical trial with a unique third, masked choice extension phase for 45 men with androgen deficiency-type symptoms in energy and sexual domains but without pathologic hypogonadism. In the third phase the participants, remaining blinded to their original treatment, chose which of the two previous treatment they regarded as better for the third phase. Despite adequate power, the participants reported no significant benefit of testosterone treatment nor could distinguish it from placebo more than by chance. For details see Mok et al. (66).

The modest magnitude of the benefits of testosterone in ageing men without pathologic hypogonadism is also illustrated in the Testosterone for Androgen Deficiency-Like Symptoms (T4ADS) study (66). In this study men with non-specific symptoms resembling those ascribed to androgen deficiency and seeking testosterone treatment were randomized to daily testosterone gel administration in a three-way, placebo-controlled cross-over study (**Figure 3**). Beyond the conventional two-way cross-over, this study featured a novel third, choice extension arm. In that third arm the participants, while remaining masked to the sequence of their two previous treatments, chose which previous treatment they preferred as more effective to continue into the third arm while remaining still masked. Overall testosterone treatment showed no benefits for energy or sexual function symptoms or a wide variety of quality-of-life measures compared with placebo. Strikingly, although baseline testosterone was a significant covariate associated with study endpoints (energy, sexual symptoms), neither endpoint was significantly improved by testosterone more than by placebo treatment. This indicated that reduced blood testosterone as a covariate reflected only indirect effects presumably due to underlying non-reproductive disorders. Finally, men with these non-specific androgen deficiency-like symptoms could not distinguish testosterone from placebo treatment for symptom relief more than by chance.

Such minimal symptomatic benefits of testosterone for men without pathologic hypogonadism may explain the high discontinuation rates of men with only non-specific symptoms (but not impaired intrinsic reproductive function) who start treatment with testosterone gels for “hypogonadism”, by the expansive redefinition (67, 68). Undeterred enthusiasts may rationalise such frequent discontinuation of useless testosterone by claiming that at least early discontinuation minimises potential harm. However, providing useless, wasteful, and possibly harmful treatment of patients benefits solely testosterone product sales and not those patients. Furthermore, complacent endorsement of poor management tacitly accepts a debasing of responsible prescribing, undermining trust, and confidence in medical practice. Vaccination hesitancy in the COVID pandemic is a reminder that trust in medical professionalism is a critical public health resource. Such tacit acceptance of suboptimal medical management subtracts from that trust. Instead, there is a need for sensible action to curb this problem and maintain medical credibility and integrity, the first step being recognition and awareness.

An ironic, alternative perspective on this issue derives from the well-established findings that supraphysiological testosterone or synthetic androgen dosage can achieve dose-dependent increases in muscle mass and strength from below to well above the natural physiological limits in healthy eugonadal men (69–72). One consequence of the confusion created by deforming the term “hypogonadism” is the implication that testosterone treatment for male ageing should be used solely within the limits of conventional physiological replacement therapy. This stricture has been invoked mainly to circumvent the FDA and other regulatory agencies requirement for establishing sound clinical efficacy and safety through adequately powered, randomized, placebo-controlled clinical trials. It could instead be considered whether supraphysiological doses of testosterone or of synthetic androgens (including the new category of SARMs) could be evaluated through rigorous clinical trials. That is the rationale for synthetic androgen use as pharmacologic therapy in non-gonadal disease (73) which, even though often superseded by more specific and expensive modern therapeutics, remains an effective (and cost-effective) therapeutic alternative. It is possible to consider whether deliberately pharmacological, supraphysiological testosterone doses may be more effective than what has been evaluated so far.

## Safety

There is no convincing evidence for the safety of testosterone treatment of men without pathologic hypogonadism. Without such data, testosterone treatment cannot be recommended. There are at least three well defined major areas of public health concern about the unknown safety impact of testosterone treatment on male ageing; others may yet appear. These major issues comprise risks of testosterone-initiated or accelerated cardiovascular and prostate health and androgen dependence.

### Cardiovascular Safety

In the absence of pathologic hypogonadism, clinical studies of safety for testosterone treatment for cardiovascular events [including heart failure (74) or venous thromboembolism (75, 76)] are reported as either observational cohorts (75, 77–81) or meta-analyses of short-term placebo-controlled randomized clinical trials (74, 77, 82–90). The former lack cogency due to the intractable confounding of cardiovascular disease mechanisms with numerous risk factors having comparable or greater influence than testosterone as well as the inability of observational data alone to justify interventional treatment. Similarly, short-term placebo-controlled trials lack the decades of exposure required to convincingly appraise the risks of testosterone treatment on cardiovascular disease. These meta-analyses have realistically summarised the component studies as being of very low quality (82) and substantially underpowered (90). They also identified additional new confounding factors which have not been widely incorporated into the meta-analyses. These include transient early adverse effects rather than stable, sustained effects (75, 83), statin effects on blood testosterone (91), DHT produced by testosterone administration (84) and industry sponsorship reporting fewer adverse effects (86). These conflicting and inconclusive meta-analyses of adverse cardiovascular effects derived from the same set of testosterone treatment trials have led the FDA to mandate a prospective interventional cardiovascular safety study (TRAVERSE). TRAVERSE aims to recruit 6000 men without pathologic hypogonadism to be randomized to daily testosterone or placebo gel for up to 5 years and is scheduled to complete in 2022. Whether the exposure time is sufficient to identify any long-term cardiovascular events remains to be established.

### Prostate Safety

Another concern for the long-term safety of testosterone treatment of men without pathologic hypogonadism is whether it will increase the risk of testosterone-induced or accelerated benign or malignant prostate disease. Sustained exposure to adult male blood testosterone concentration from puberty onwards is required for completion of prostate development, a necessary but not sufficient condition for the development of late-life prostate diseases. Available meta-analyses of observational studies suggest minimal risk of subsequent prostate cancer associated with exposure to endogenous or exogenous testosterone exposure over many years of observation (92, 93). Similarly, pooling available randomized, placebo-controlled clinical trials of exogenous testosterone also showed no measurable risk of subsequent prostate cancer; however, exposure was only for up to 3 years, far shorter than the decades-long latency of prostate diseases (94).

The prevalence of benign prostate disease increases steeply with age with a majority of men over age 50 years having benign prostate hyperplasia (95–98). Hence these very frequent disorders invoke large medical care costs for an ageing population. The impact of exogenous testosterone treatment is so far confined to studies of symptomatic effects for up to 3 years (99–101) but so far there are no epidemiological studies on the effects of testosterone treatment on the prevalence of benign prostate diseases of older age.

Consequently, population surveillance of prostate diseases is needed to detect any impact of the recent epidemic of testosterone prescribing. For prostate cancer, this requires making the distinction between screened-detected, organ-confined and life-threatening advanced or metastatic cancers. Due to the much longer disease latency, it is doubtful that the TRAVERSE study can evaluate safety for the risk of testosterone-induced or accelerated prostate disease. Hence for both benign and malignant prostate diseases of older men, there is minimal evidence that testosterone will have no adverse effects on prevalence of late-life prostate diseases.

### Iatrogenic Androgen Dependence

**Figure 4:**
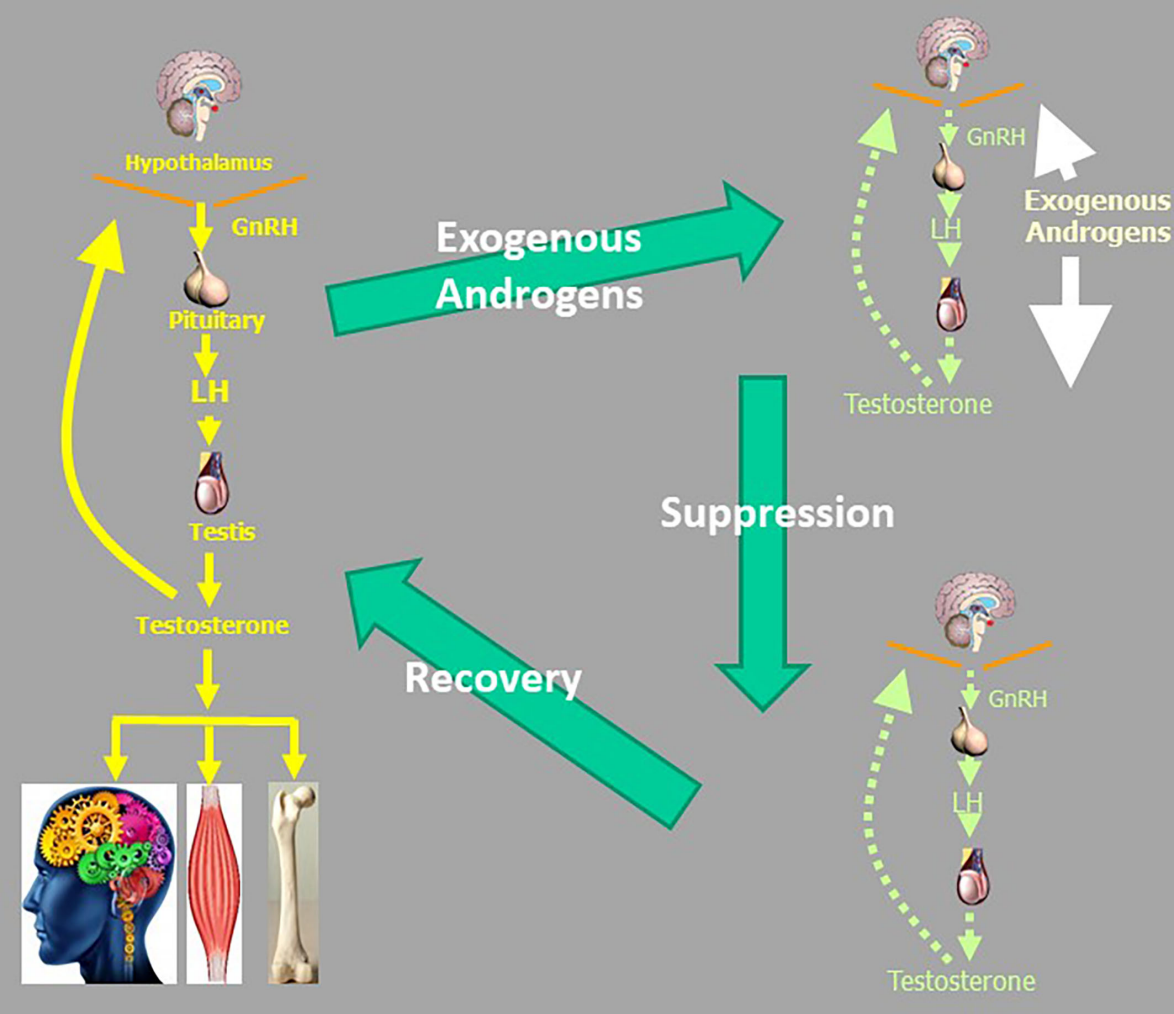
The vicious circle of androgen abuse which can lead to a cycle of androgen dependence. Suppression of the male reproductive axis due to exogenous androgens recovers slowly over many months leaving iatrogenic androgen deficiency withdrawal symptoms which encourage resuming androgen use to alleviate androgen deficiency symptoms. Even if men did not have genuine not androgen deficiency before starting exogenous testosterone or other androgens, they may be rendered androgen deficient after they cease exogenous androgen intake.

A further and less considered public health concern is the creation of iatrogenic androgen dependence when testosterone treatment is administered to men without reproductive pathology (102). In men with pathological hypogonadism, the irreversible underlying reproductive system disorders require life-long testosterone replacement as none of these conditions are reversible or rectifiable. In contrast, administration of testosterone to men with normal reproductive system suppresses endogenous testosterone production due to androgenic negative feedback (**Figure 4**). When testosterone administration to men without pathologic hypogonadism ceases, this leads to withdrawal symptoms from transient androgen deficiency until the hypothalamo-pituitary unit axis recovers, which may take weeks to months (103–105). The determinants of slow recovery from suppression of the HPT axis remain to be fully clarified but duration and dose of exogenous androgen exposure appear to be important factors. When invoked, such withdrawal symptoms can lead to resuming testosterone administration to alleviate the iatrogenic androgen deficiency, creating a vicious circle of androgen dependence. For example, when testosterone was no longer subsidized by the Australian national health scheme in 2015, a switch to private, non-subsidised testosterone prescribing occurred maintaining the same overall rate of testosterone usage. This indicated that men wishing to stop futile testosterone treatment had difficulty discontinuing most likely due to seeking symptom alleviation of iatrogenic androgen deficiency withdrawal symptoms by resuming testosterone misuse (102). While this cycle of dependency is most overt among androgen abusers, the same underlying mechanism are operative among men without pathologic hypogonadism who initiate unjustified testosterone treatment (102). This creates a dilemma of “tail chasing” whereby administering salvage testosterone leads to further accentuating of the underlying HPT axis suppression making eventual withdrawal even harder, slower, and less successful. These considerations invalidate the complacent view that testosterone misuse is simply a harmless hobby.

## Managing the Demand for Unjustified Testosterone Treatment

It is not easy in the present environment for endocrinologists to avoid being drawn, however reluctantly, into testosterone misuse. Many endocrinologists are referred patients with a single, marginally low blood testosterone measurement seeking testosterone treatment for “hypogonadism”. Under the misguidance of numerous extant guidelines or other manifestos, encourage excessive testosterone prescribing where there is any clinical doubt, which is almost always. Like family doctors or GPs, they may fear that if they do not succumb to prescribing on demand, the patient will go doctor shopping and get the testosterone they think they need or demand elsewhere. These dilemmas are enlivened, if not enlightened, by concerted marketing and papers emanating from pharma, upscale single-issue men’s health clinics and academic enthusiasts. These rarely highlight the vested commercial interests, where present, promoting testosterone use outside approved indications under the disease-mongering rubric of “hypogonadism”.

Testosterone is unique among hormones for its high level of public recognition, which unfortunately is imbued with fantasies and fictions unrelated to endocrine reality, an enchantment that easily unlocks latent but irrational wishes for rejuvenation. Reproductive medicine is unique in that, unlike other medical specialties, virtually everyone’s personal experience of sex and reproduction provides them with the subjective confidence they possess sound insight into reproductive biology and medicine without needing recourse to the established objective facts. This particularly extends to beliefs about what testosterone is and does biologically. This illusion of sophisticated expertise forms a powerful coupling with tenacious wishful thinking. This latent demand is readily entrained by clever marketing from pharma and other commercial enterprises that promotes testosterone’s use as an anti-ageing or sexual dysfunction tonic, reinforced by cyberchondria. The net effect is to expand the horizons of prescription drug misuse from opiates and benzodiazepines to include testosterone. Under the disease-mongering expanded definition of “hypogonadism” (1), as cover for legitimating testosterone prescribing on demand and/or for treating trivial clinical and/or biochemical features unrelated to pathologic disorders of the HPT axis created a 100-fold increase in expenditure on prescription testosterone products over three decades to 2010 (1).

The remedies for this dire situation start with reinstating the proper definition of hypogonadism and rejecting the permissive, elastic expansion of its boundaries and unjustified screening for low blood testosterone. Testosterone misuse derives from prescribing testosterone in an inappropriate clinical context without pathologic hypogonadism. Hypogonadism is and has always been a clinical diagnosis with a pathological basis, confirmed by hormone assays – not the other way around. Excessive prescribing of off-label testosterone is, like the prescription drug misuse of benzodiazepines and opiates, easy for busy doctors to be swayed into as giving patients something to go away with, apparently harmless prescribing on demand without concern about wider implications for the patient let alone society. This coincides with the testosterone marketing strategy of the early years of the 21^st^ century whereby targeted efforts aimed to shift testosterone prescribing onto family doctors or GPs, but away from endocrinologists. However, busy family doctors or GPs who find it easy to write prescriptions giving patients something they go away with, but those doctors are less familiar with the critical basis for, and consequences of, initiating testosterone treatment in the absence of genuine hypogonadism. Reversing this misdirection for the initiation of testosterone prescribing away from specialists was one of the key reforms created by the 2015 reimbursement restrictions of Australia’s Pharmaceutical Benefits Scheme which required a specialist to initiate new testosterone treatment though, once started by a specialist, it could be continued by a family doctor or GP (102, 106).

Doctors, especially endocrinologists, should be vigilant in recognizing that testosterone is highly susceptible to wishful thinking and marketing fueled by confected, internet-driven patient fantasies. They should take care to distinguish between pathological hypogonadism and functional disorders leading to reduced blood testosterone, a functional pathophysiological state, not a disease. Ageing co-morbidities including the pseudo-hypogonadism of obesity and functional impacts on the HPT axis from numerous systemic diseases causing mild lowering of blood testosterone, should not be confused with genuine organic hypogonadism. Care is needed when considering marketing-friendly, disease-mongering guidelines that promote excessive testosterone prescribing that contribute to the epidemic of excessive testosterone prescribing of recent decades. When termed “hypogonadism” (or any of its neologistic synonyms) under the expanded disease-mongering definition, these ageing-comorbidities are a fiction in search of a definition. Finally, there is no basis for population or individual patient screening (“case finding”) by measuring blood testosterone without a genuine clinical suspicion of underlying male reproductive pathology based on the clinical presentation including examination of testes. When measurement of blood testosterone is justified by the clinical presentation including physical examination, it should be accompanied by blood LH, FSH and SHBG to clarify interpretation and assays should be performed multiple times. Pathologists should be encouraged to switch to more accurate LCMS-based measurements of testosterone and stop reporting the misleading imaginary fractions of testosterone (“free”, “bioavailable”), a numerical artifice signifying nothing for clinical guidance (41).

## Conclusion

Testosterone prescribing for men without pathological hypogonadism is a therapeutic illusion in search of a definition. It is fostered by wishful thinking of an affluent populace with eyes mistily focused on the mirage of rejuvenation. Although cultivated by the disease-mongering expanded definition of “hypogonadism” as any lowering of blood testosterone coupled with any non-specific complaint, it is nowhere near justified by current standards of efficacy and safety evidence. As such, testosterone treatment other than replacement therapy for authentic hypogonadism should be confined to adequately powered, well-designed and placebo-controlled clinical trials geared to determining the efficacy and safety of testosterone prescribing for functional states, including but not limited to, age-related hypogonadism. Available evidence gives minimal grounds for expecting such proof in the foreseeable future. In the meantime, doctors need to be supported resisting the popular but misguided demand for testosterone. In many respects this is an educational challenge like smoking cessation, which was equally driven by clever advertising confecting public demand for the product without health benefit.

The public health consequences of the recent epidemic-like increase in testosterone prescribing on cardiovascular and prostate health and iatrogenic androgen dependence remain to be evaluated over coming decades. At best it may have little adverse impact but there could be detrimental changes in cardiovascular and/or prostate health. It may be claimed in defence of unjustified off-label testosterone prescribing that men treated with testosterone for no good reason often stop treatment after a short period. While short-term evidence suggests that this is true, it comes at a cost of undermining the credibility and confidence in the integrity of medical care. Furthermore, some evidence suggests that significant numbers of men who start testosterone treatment may have difficulty stopping it even if it proves ineffective as they become androgen dependent from androgen deficiency withdrawal symptoms while their endogenous testosterone production resumes, albeit slowly. In any case this is an indictment on the judgement of those prescribing T without proper evidence-based indication and the potential harm and waste this creates. Adverse outcomes would grievously erode public confidence in medical integrity and might eventually curb doctors’ traditional freedom to prescribe any registered drug off-label. The permissive disease-mongering redefinition of “hypogonadism”, an elastic broadening of the diagnosis facilitates much more testosterone prescribing resulting in a deterioration in clinical evaluation of androgen status creating a churn market in testosterone products and disappointment in misled patients. While this may favour pharma and other commercial enthusiasts with self-serving benefits, this misadventure is a net loss for the highest standards of medical care in the 21^st^ century.

## Author Contributions

The author confirms being the sole contributor of this work and has approved it for publication.

## Conflict of Interest

The author declares that the research was conducted in the absence of any commercial or financial relationships that could be construed as a potential conflict of interest.

## Publisher’s Note

All claims expressed in this article are solely those of the authors and do not necessarily represent those of their affiliated organizations, or those of the publisher, the editors and the reviewers. Any product that may be evaluated in this article, or claim that may be made by its manufacturer, is not guaranteed or endorsed by the publisher.
